# Supplementary material for: Building health: Motivational factors to enhance systematic work environment management in the construction industry
Source: PLoS One. 2025 Oct 6;20(10):e0333178. doi: 10.1371/journal.pone.0333178 (PMC12500124; doi:10.1371/journal.pone.0333178)
Supplement: Attachment — Submitted filename: New Microsoft Word Document.docx [file pone.0333178.s001.docx]

**Building health: motivational factors to enhance systematic work environment management in the construction industry**

(*) Overall, this document should be worked on to make necessary adjustments regarding academic writing/phrasing.

85, 94, 197 – Authors should avoid using “we,” “I,” or “our”, etc. in communicating the research process or findings.

97, 98 – You need to systematically introduce the elements of a participative intervention in this study section before indicating the purpose of examining how they foster a well-functioning and continuous SWEM in small and medium-sized construction. This will help create a basis for examining the elements of a participative intervention,

198, etc. – Check your first mention of DTs and others to see if the actual words were indicated before stating acronyms. Please check the manuscript thoroughly and amend it accordingly. Besides, you need to reduce the use of acronyms significantly.

243 – 246 – Your aim can be combined and indicated in a paragraph as against in bullets.

**Methods**

Authors should indicate how they generated their interview questions.

You have not indicated a process of Themes. What are your workable themes? Hence, how did you decide to implement a semantic inductive thematic analysis?

This section needs some professional layout. It is not robust and procedural, which is the highlight of a qualitative research methodology.

**Results**

As indicated earlier, you did not indicate your working themes. Hence, reporting interviews in themes you did not indicate is strange.

Besides, all reported statements of respondents (interviewees) should be in italics.

**Discussion**

Further to your discussions, authors should systematically infer their findings under each theme indicated and relate them to previous scholars’ conclusions.

This will serve as the achievement of the aim of the study about the themes.

**Conclusions**

Furthermore, your conclusion should start with the aim of the investigation, followed by a summary of your results.

Besides, this section indicates the implications of your findings to practice. This could further generate some suggestions.

**References**

Please cross-check your reference list to ensure they are in the correct format. Besides, check the reference list with your in-text citations.
