# Supplementary material for: Building health: Motivational factors to enhance systematic work environment management in the construction industry
Source: PLoS One. 2025 Oct 6;20(10):e0333178. doi: 10.1371/journal.pone.0333178 (PMC12500124; doi:10.1371/journal.pone.0333178)
Supplement: Attachment — Submitted filename: Response_to_Reviewers.docx [file pone.0333178.s003.docx]

Response to Reviewer

Thank you for your valuable comments on the current text. They have been helpful in the work of revising the article. The following section presents a summary of the changes that have been implemented in response to the provided feedback.

| **Reviewer comment** | **Changes made** |
| --- | --- |
| 85, 94, 197 – Authors should avoid using “we,” “I,” or “our”, etc. in communicating the research process or findings. | The use of personal pronouns in the text has been adjusted according to the reviewer's comments, and all personal pronouns have been removed. |
| 97, 98 – You need to systematically introduce the elements of a participative intervention in this study section before indicating the purpose of examining how they foster a well-functioning and continuous SWEM in small and medium-sized construction. This will help create a basis for examining the elements of a participative intervention, | The term "purpose" has been removed, and the text has been revised to a more general form that addresses the subject of the study. Additionally, elements of participatory interventions have been omitted. |
| 198, etc. – Check your first mention of DTs and others to see if the actual words were indicated before stating acronyms. Please check the manuscript thoroughly and amend it accordingly. Besides, you need to reduce the use of acronyms significantly. | A thorough review of all acronyms has been conducted to ensure the use of relevant concepts prior to the introduction of each acronym. To significantly reduce the prevalence of acronyms, the following modifications have been implemented. The acronym "DT" has been replaced with "the team," "WS" with "workshops," and "MI" with "motivational interviewing." The utilization of the acronym "SWEM" has been changed in two ways. First, the phrase "Systematic Work Environment Management" has been used in a few places at the beginning of the text. Otherwise, SWEM has been replaced with “OSH management.” |
| 243 – 246 – Your aim can be combined and indicated in a paragraph as against in bullets. | The aim has been revised in accordance with the reviewer's recommendation. |
| **Methods**  Authors should indicate how they generated their interview questions.  You have not indicated a process of Themes. What are your workable themes? Hence, how did you decide to implement a semantic inductive thematic analysis?  This section needs some professional layout. It is not robust and procedural, which is the highlight of a qualitative research methodology. | The text now provides a comprehensive explanation of the rationale behind the choice of an inductive approach for the thematic analysis. The text has also been supplemented with an expanded and corrected description of the analysis process used to develop themes from the empirical data. The themes are clearly outlined. Regarding how the interview questions were generated, the text has been supplemented with background information and links to relevant previous research in the field that influenced the content and structure of the interview questions. |
| **Results**  As indicated earlier, you did not indicate your working themes. Hence, reporting interviews in themes you did not indicate is strange.  Besides, all reported statements of respondents (interviewees) should be in italics. | As previously addressed in response to the reviewer's comments under methodology, the text has also been expanded and corrected to include a clearer description of the analysis process used to identify themes from the empirical data. The themes are clearly outlined.  All statements of the respondents are now in italics |
| **Discussion**  Further to your discussions, authors should systematically infer their findings under each theme indicated and relate them to previous scholars’ conclusions.  This will serve as the achievement of the aim of the study about the themes. | Based on the reviewers’ comments, the discussion section has been thoroughly and carefully rewritten. The results have been systematically linked to each identified theme. Additionally, where relevant, the results have been contextualized with conclusions drawn from previous research. |
| **Conclusions**  Furthermore, your conclusion should start with the aim of the investigation, followed by a summary of your results.  Besides, this section indicates the implications of your findings to practice. This could further generate some suggestions. | The conclusions now begin with the aim. The conclusion section has been revised and has also been provided with suggestions for practical implications |
| **References**  Please cross-check your reference list to ensure they are in the correct format. Besides, check the reference list with your in-text citations. | The reference list was reviewed, and it appears there was an issue with the EndNote reference management system, resulting in 10 references being omitted. The reference list has now been corrected, and new references have also been added based on the revised text. |
